# Case Report: Reconstruction of the Right Atrium With the Left Atrial Appendage

**DOI:** 10.3389/fcvm.2021.782235

**Published:** 2021-11-22

**Authors:** Xuan Jiang, Jinduo Liu, Yuhai Zhang, Tianxiang Gu, Bo Liu

**Affiliations:** Department of Cardiac Surgery, First Affiliated Hospital, China Medical University, Shenyang, China

**Keywords:** infective endocarditis, right atrium, left atrial appendage, heart surgery, infection

## Abstract

We herein present a case of infective endocarditis of the mitral valve and a paravalvular abscess around the tricuspid valve. Preoperative blood culture confirmed the presence of pathogenic diphtheroids. During the operation, an unexpected infection of the free wall of the right atrium (RA) near the tricuspid annulus was found. We harvested the left atrial appendage (LAA) en bloc. After resection of the infected and abnormal tissues, the resected LAA was used to reconstruct the RA. The infected mitral valve was replaced with a mechanical valve without any accident. Postoperative echocardiography showed that the RA had a supple shape, with no kinking.

## Introduction

Right-sided infective endocarditis rarely causes invasive complications such as abscesses or pseudoaneurysms (1). This can be explained by the lower pressure and lesser amount of muscle tissue around the tricuspid valve (TCV). However, the right atrium (RA) can become infected or can be invaded by tumors. In this case, the RA can be reconstructed using autologous pericardium, bovine pericardium, or an extracellular-matrix patch (ECM) (2). Herein, we present a case of paravalvular abscess around the TCV. We resected the infected RA and reconstructed it with the left atrial appendage (LAA).

## Case Description

A 25-year-old female patient presented to the Cardiac Surgery Department with intermittent fever and dyspnea that had lasted 6 months. She had no history of intravenous drug use. On the initial visit to Cardiac Surgery, her body mass index (BMI) was 25.1. Her respiratory rate was normal, but she had hypertension (blood pressure [BP] 180/90 mmHg). The electrocardiogram showed sinus rhythm. This patient had a moderate systolic murmur at the apex of the heart. She received telmisartan to control her BP. Serum creatinine (sCr) was 157 μmol/L. Her post-admission blood culture was positive, confirming that Corynebacterium diphtheriae was the pathogen. Transthoracic echocardiography (TTE) showed multiple small vegetations on the surface and tip of the mitral valve, mitral valve regurgitation, and mild TCV regurgitation (Supplementary Video 1). The primary diagnoses were infective endocarditis and renal dysfunction. TTE, computed tomography, and magnetic resonance imaging showed no involvement of the right atrium.

**Figure 1:**
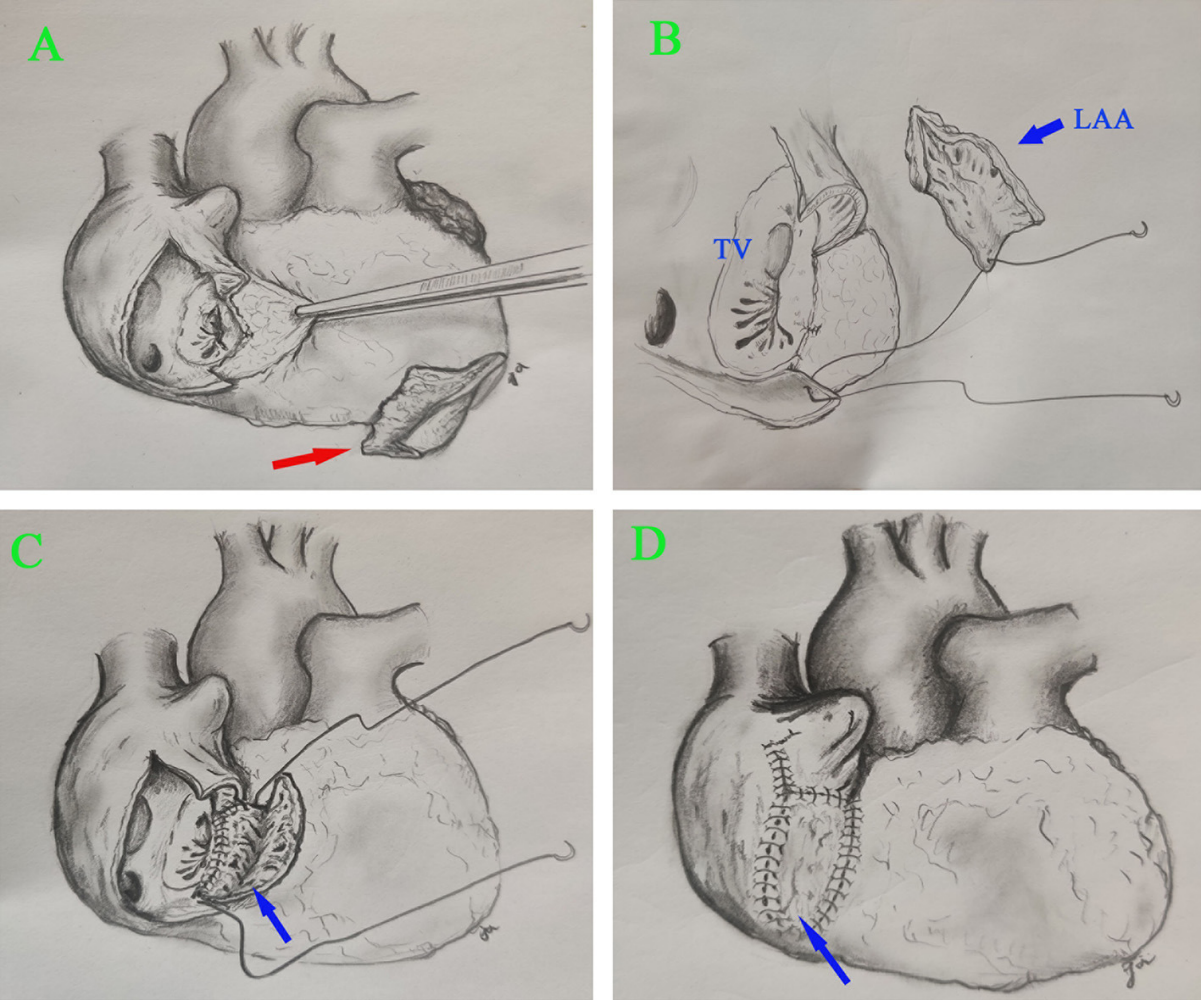
Surgical view of the reconstructed RA. **(A)** Resection of infected RA tissue. **(B,C)** RA reconstruction using autologous LAA. **(D)** The reconstructed RA. Right arrow, infected RA tissue; blue arrow, LAA.

After broad-spectrum empirical antibiotic treatment (0.5 g imipenem/cilastatin 3 times a day, and 0.5 g vancomycin 2 times a day) for 1 week, the patient had no fever but was suffering from progressive heart failure. We performed transthoracic echocardiography again and found that the vegetation was stable, but the mitral regurgitation had progressed. To avoid progressive heart failure, we decided to perform early mitral valve replacement using a median sternotomy. Cardiopulmonary bypass (CPB) was established in the standard manner. We cross-clamped the aorta, and we arrested the heart using antegrade cold del Nido cardioplegia. The appearance of the RA during the operation was normal (Supplementary Video 2). After the RA was opened, the free wall thereof near the tricuspid annulus was found to contain infected tissue and to have formed abscesses. Part of the tricuspid annulus was also infected. We first attempted to remove the partially infected tissue and then examined the LAA, which we harvested en bloc. The left atrium looked normal without any infection, so we did not conduct tissue culture or histological examination. We found three yellow and dark-red vegetations (about 1 × 1 cm each) on top of the mitral valve, which was also infected. We replaced this valve with a mechanical valve (size #25; St. Jude Medical [Abbott Laboratories, Abbott Park, IL, USA]). After resecting all infected and abnormal tissues of the RA free wall, we observed insufficient RA tissue to permit a safe closure, so we instead performed circumferential reconstruction of the RA. The area of infected tissue was about 4 × 3.5 cm, and it was close to the inferior vena cava and right atrioventricular groove (Figure 1; Supplementary Video 2). Part of the infected tricuspid annulus was also removed during the operation. We sutured the remnant tricuspid valve and annulus with a 5-0 PROLENE suture (Johnson & Johnson Medical Devices, New Brunswick, NJ, USA). The resected LAA was used to reconstruct the RA from the atrioventricular junction to the inferior vena cava using another 5-0 PROLENE suture. The autologous atrial tissue was pliable and easily handled. The saline test showed mild TCV regurgitation.

**Figure 2:**
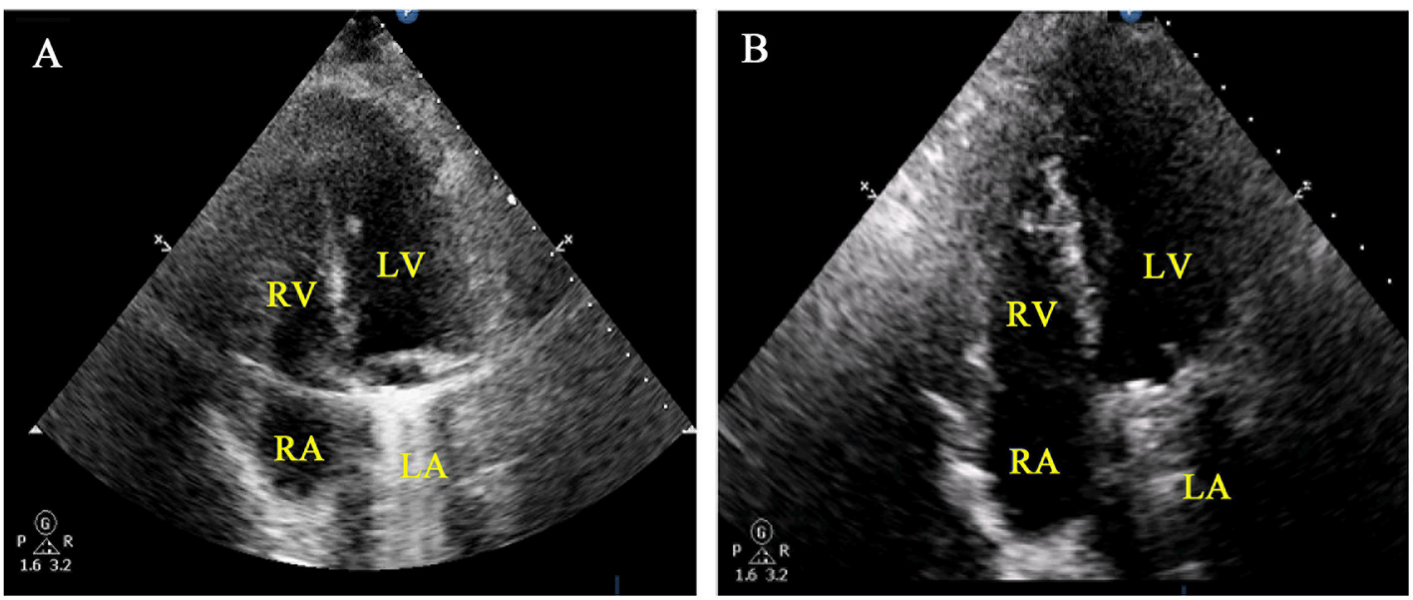
Post-operative findings in a patient with infective endocarditis and paravascular abscess of the RA. **(A)** Immediately postoperative TTE of the RA and TCV. **(B)** TTE showed no shrinkage of the reconstructed RA 6 months after surgery.

The patient was weaned off CPB successfully with no inotropic support. No bleeding occurred at the suture lines of the reconstructed RA. CPB time was 105 min, and aortic cross-clamp time was 75 min. TTE performed immediately after surgery showed that the reconstructed RA had a good shape (Figure 2A). Postoperative chest drainage was 235 mL over a 24-h period; drainage tubes were removed on day 2 post-surgery. The patient made a good recovery and was discharged 14 days post-surgery after standard antibiotic therapy.

Histology of the resected RA confirmed myocardial tissue infection and inflammation. Diphtheroids were cultured from the removed RA and mitral valve. At 6 months post-surgery, TTE showed good remodeling of the RA without kinking or shrinkage due to fibrosis (Figure 2B; Supplementary Video 1). Heart function was good. One month after surgery, the patient returned to work. Over 9 months of follow-up, she has had no fatigue or dyspnea.

## Discussion

We herein present a rare case of insufficient cardiac tissue for an attempt at directly closing the remnant RA. Selecting suitable tissue to reconstruct the RA is difficult. Previous studies have reported the use of different patches, such as porcine and bovine pericardia (2, 3), but such exotic tissues can cause an inflammatory reaction as well as fibrosis and scar formation. Fibrosis or scars in atrial tissue may be the source of arrhythmia because it promotes conduction slowdown, blockage, and reentry. ECM might be a better choice, but it is expensive and not available in developing countries (4). In addition, it is reputed to be biologically degradable. The autologous pericardium is a better choice for the construction of the RA after tissue excision.

We speculate that using autologous atrial tissue might have several advantages. Increasing the compatibility between the grafted and original tissues can prevent some scar tissue formation. The LAA is softer and more flexible than bovine or porcine pericardial tissue. Bleeding is more easily prevented when the patches have good adaptive margins with the surrounding remnant RA tissue. In this case, we needed to reconstruct the RA tissue around the right atrioventricular groove from the endocardium to the epicardium. The shape of the LAA on two planes was suitable for the reconstruction of the RA. The removed LAA is alive and maybe infection resistant and non-thrombogenic. An autologous graft does not induce an immunologic response. The use of autologous tissue also preserves the capacity to self-repair, adaptively remodel, and grow (5).

The LAA contributes up to 8–9% of left atrial volume and is highly variable in its morphology. It is also known to have an important effect on the release of atrial natriuretic peptide (ANP). The role of LAA in triggering and maintaining atrial fibrillation (AF) has received increasing attention, especially in patients with persistent AF or AF recurrence after repeated ablation (6). The complex anatomy and cellular structure of LAA may also predispose to arrhythmia because it promotes conduction slowdown, blockage, and reentry (7). In a retrospective study of 987 patients undergoing repeat catheter ablation for AF, 27% were found to have triggers in LAA (6). Not only is LAA a significant source of AF, but that electrical isolation or resection may have an important role in the treatment of persistent AF to avoid AF recurrence. Thrombosis is more likely to occur in the LAA than in other parts of the left atrium. Patients with left ventricular dysfunction or elevated left ventricular end-diastolic pressure might also be at risk of LAA thrombosis, even without AF (8). In our case, it was appropriate to remove the LAA and preserve its endocrine function in the reconstructed RA. However, the continuation of the endocrine function of the LAA after reconstruction still needs further exploration.

A positive blood culture confirms that Corynebacterium diphtheriae is the pathogen causing severe endocarditis in this case. Corynebacterium diphtheriae endocarditis is considered a rare disease that affects heart valves and seems to be very virulent and destructive. It is possible to select patients requiring emergency surgery based on the underlying valve pathology. Patients with abnormal or prosthetic valves should be prepared for emergency surgery if necessary (9). Surgery must include extensive debridement of all infected tissue, similar to that necessary for *staphylococcal endocarditis*.

Reconstruction of the RA carries the risk of several complications: bleeding, deformity, kinking, and degeneration. Our method is simple and economical, avoiding such complications. The size of the LAA is the main limitation of this method.

## Data Availability Statement

The original contributions presented in the study are included in the article/Supplementary Material, further inquiries can be directed to the corresponding authors.

## Ethics Statement

The studies involving human participants were reviewed and approved by China Medical University. The patients/participants provided their written informed consent to participate in this study. Written informed consent was obtained from the individual(s) for the publication of any potentially identifiable images or data included in this article.

## Author Contributions

All authors listed have made a substantial, direct and intellectual contribution to the work, and approved it for publication.

## Funding

This study was supported by Liaoning Education Department. The grant number is XLYC201802066. Funding receipt is TG.

## Conflict of Interest

The authors declare that the research was conducted in the absence of any commercial or financial relationships that could be construed as a potential conflict of interest.

## Publisher's Note

All claims expressed in this article are solely those of the authors and do not necessarily represent those of their affiliated organizations, or those of the publisher, the editors and the reviewers. Any product that may be evaluated in this article, or claim that may be made by its manufacturer, is not guaranteed or endorsed by the publisher.
